# Noncoding RNAs in pyroptosis and cancer progression: Effect, mechanism, and clinical application

**DOI:** 10.3389/fimmu.2022.982040

**Published:** 2022-08-17

**Authors:** Menghui Zhang, Pengyuan Dang, Yang Liu, Bingbing Qiao, Zhenqiang Sun

**Affiliations:** ^1^ Department of Hepatobiliary and Pancreatic Surgery, The First Affiliated Hospital of Zhengzhou University, Zhengzhou, China; ^2^ Department of Colorectal Surgery, The First Affiliated Hospital of Zhengzhou University, Zhengzhou, China; ^3^ Department of Radiotherapy, Affiliated Cancer Hospital of Zhengzhou University, Henan Cancer Hospital, Zhengzhou, China

**Keywords:** pyroptosis, non-coding RNA, cancer, tumor resistance, tumor immunity

## Abstract

Cell death is generally classified into two categories: regulated cell death (RCD) and accidental cell death (ACD). In particular, RCD is a kind of genetically controlled process, including programmed apoptotic death and programmed necrotic death. Pyroptosis, an inflammatory form of programmed necrotic death, causes inflammation in cells. The influence of pyroptosis on tumor is complicated. On the one hand, pyroptosis triggers antitumor response. On the other hand, pyroptosis may induce carcinogenesis. Pyroptosis is initiated by various factors, especially non-coding RNAs. In this review, we discuss the effects of ncRNAs on pyroptosis and the mechanisms by which ncRNAs initiate pyroptosis. Moreover, we introduce the influence of ncRNA on tumor resistance *via* pyroptosis. Additionally, we summarize how ncRNA-associated pyroptosis modulates the tumor microenvironment (TME) and thereafter triggers antitumor immune response. Finally, pyroptosis-related ncRNAs are promising diagnostic and immunotherapeutic biomarkers and therapeutic targets

## Highlights

- NcRNA-regulated pyroptosis is a double-edged sword for tumor development.
- NcRNAs regulate tumor resistance *via* pyroptosis and could be potential therapeutic targets to overcome tumor resistance.
- NcRNAs trigger pyroptosis, causing fierce inflammatory response and remodeling TME.
- Pyroptosis-related ncRNAs are expected to serve as biomarkers of diagnoses and immunotherapeutic efficiency.

## Open questions

- The influence of ncRNA-regulated pyroptosis is complex on tumor and requires further experimentations.
- There is still a lack of a precise pyroptosis-related drug delivery platform to specifically trigger pyroptosis in extracellular milieu in tumor sites.
- The factors leading to the expression change of pyroptosis-associated ncRNAs within a cancerous context are unknown.
- The mechanisms by which circRNAs are sensed in the pyroptotic pathway are unclear.

## Pyroptosis

### The history and characteristics of pyroptosis

In 1992, Lindgren, S. W. firstly described pyroptosis in *Shigella flexneri*-infected macrophages (1). Pyroptosis is the inflammatory programmed necrotic death that is characterized by cell lysis, cell content release, membrane rupture, DNA fragmentation, and an intact nucleus (2–7). Rapid death speed and huge morphological change distinguish pyroptotic cells from apoptotic cells (8).

Programmed necrotic death is generally categorized as necroptosis, ferroptosis, and pyroptosis. Evolutionarily, viruses attained the ability to evade their host’s defense and the immune system (9, 10). Necroptosis is thought to be conserved as an MLKL-mediated necrosis to defend viruses during evolution (11). Ferroptosis is a kind of iron-dependent necrosis by lipid peroxidation. Unlike necroptosis and pyroptosis, ferroptosis is not induced by classic signaling, such as through a death receptor or a DNA sensor. Additionally, it is not comprehensively clarified how the plasma membrane might rupture in ferroptosis. Furthermore, this necrotic-type cell death, ferroptosis, occurs in a non-cell-autonomous manner referred to as synchronized regulated necrosis (11). Given pyroptosis, it is a gasdermin-mediated necrosis to amplify inflammasome action and eventually trigger fierce inflammatory response (12–18).

The roles of pyroptosis in human diseases have emerged. Triggering pyroptosis contributes to various diseases, including infection such as *L. pneumophila* (19, 20), autoinflammatory genetic diseases such as Familial Mediterranean Fever (FMF) (21, 22), inflammation such as cytokine release syndrome (CRS) (23, 24), noninfectious diseases such as alcoholic hepatitis (25, 26), and cancers such as non-small cell lung cancer (NSCLC) (27, 28). Notably, pyroptosis is a double-edged sword for tumorigenesis and progression. LINC00958 inhibited miR-4306 levels to trigger AIM2-mediated pyroptosis pathway and promoted cancer cell survival (29). By contrast, Liu et al. reported that pyroptosis attenuates cancer cell viability and proliferation in glioma (30). Therefore, the mechanisms of pyroptosis in tumor are attracting increasing attention.

### The pathways of pyroptosis

Dying cells caused by therapeutic regimens, including chemotherapy, radiotherapy, targeted therapy, and immune therapy, activate pyroptosis in cancer (17, 31). In addition, alcohol accumulation also triggers pyroptosis in the distal esophagus (32). However, it is still unclear whether ultrasound irradiation could activate pyroptosis in extracellular matrix.

Pyroptotic cell death is mainly triggered by two pathways. In the GSDMD-mediated canonical pathway, the danger-associated molecular patterns (DAMPs) and pathogen-associated molecular patterns (PAMPs) induce and activate caspase-1 inflammasome. Thereafter, the caspase-1 inflammasome cleave GSDMD and pro-IL-1 family. GSDMD-N terminal (GSDMD-NT), the active state of GSDMD, forms a pore in cell membrane, leading to the liberation of cellular contents, including mature IL-1β, IL-18, ATP, HMGB1, and chemokines (33–35). In addition to caspase-1, caspase-4, -5, and -11 also are critical for pyroptosis activation. Caspase-4, -5, and -11 directly recognize bacterial lipopolysaccharide (35), whereas caspase-11-activating inflammasomes only lead to GSDMD cleavage without maturation of the cytokines (11, 16). In the GSDME-mediated noncanonical pathway, activated caspase-3 cleaves GSDME, which converts noninflammatory apoptosis to inflammatory pyroptosis. GSDME-NT eventually generates cell membrane pores (36, 37).

There are other GSDM family members mediating pyroptosis. GSDMA, for example, is the first GSDM family member being identified (38). However, GSDMA polymorphisms have been linked to autoimmune (39) and chronic inflammatory diseases (40). The understanding of the impact of GSDMA on cancers needs to be further explored. In addition, the overexpression of GSDMB has been found in many cancers, such as cervical, breast, gastrointestinal, and hepatic cancers, which is associated with the poor prognosis (41–44). Furthermore, GSDMC was first clarified in melanoma as a marker for progression. It was worth noting that GSDMC can be induced in breast cancer by hypoxia-triggered STAT2 phosphorylation and its complexation with nuclear-translocated programmed cell death ligand 1 (PDL1) (45).

Taken together, pyroptosis was firstly observed in infectious diseases. Furthermore, GSDM family members are the key executors of pyroptosis. As research on pyroptosis accumulated, the effects of pyroptosis were clarified deeply and comprehensively, especially on cancers. Additionally, the regulatory networks of pyroptosis were also demonstrated, among which ncRNAs have a pronounced impact on cancer cell pyroptosis and the tumor microenvironment (TME).

## Effects of ncRNAs on tumor pyroptosis

**Figure 1:**
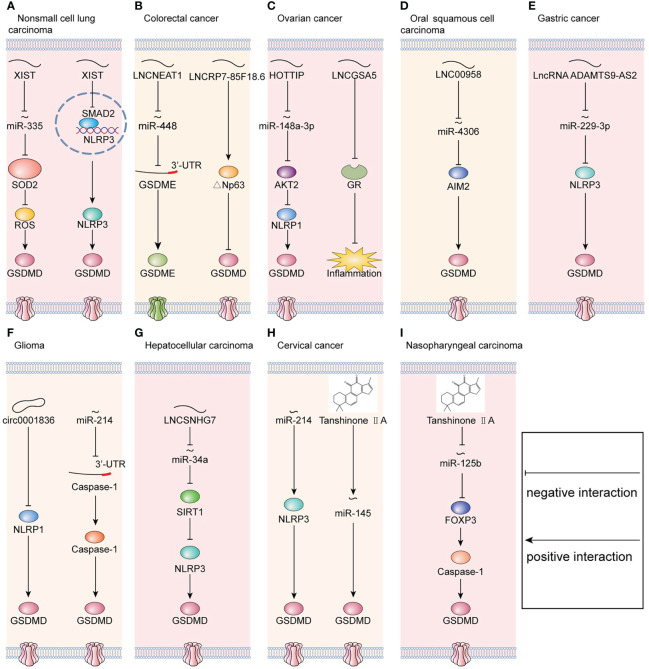
Molecular mechanisms that regulate pyroptosis in cancers. **(A)** In non-small cell lung carcinoma, LNCXIST regulates pyroptosis *via* the miR-335/SOD2 axis and SMAD2/NLRP3 axis simultaneously. **(B)** In colorectal cancer, LNCNEAT1 regulates pyroptosis *via* the miR-448/GSDME axis, and LNCRP7-85F18.6 regulates pyroptosis *via* △Np63. **(C)** In ovarian cancer, LNCHOTTIP regulates pyroptosis *via* the miR-148a-3p/AKT2/NLRP1 axis. Meanwhile, LNCGSA5 interferes with glucocorticoid receptor (GR), which blocks the upregulation of anti-inflammatory proteins and eventually promotes inflammasome formation. **(D)** In oral squamous cell carcinoma, LNC00958 regulates pyroptosis *via* the miR-4306/AIM2 axis. **(E)** In gastric cancer, LncRNA ADAMTS9-AS2 regulates pyroptosis *via* the miR-229-3p/NLRP3 axis. **(F)** In glioma, downregulation of circ0001836 promotes pyroptosis *via* epigenetically upregulating NLRP1. Meanwhile, miR-214 directly binds to the 3’-UTR of caspase-1 and consequently inhibits pyroptosis. **(G)** In hepatocellular carcinoma, LNCSNHG7 regulates pyroptosis *via* the miR-34a/SIRT1 axis. **(H)** In cervical cancer, miR-214 regulates pyroptosis *via* targeting to NLRP3. Moreover, tanshinone IIA promotes pyroptosis *via* upregulating miR-145. **(I)** In nasopharyngeal carcinoma, tanshinone IIA regulates pyroptosis *via* the miR-125b/FOXP3 axis.

For decades, in the field of cancer biology, researchers focused on the involvement of protein-coding genes. However, recently, it was discovered that an entire class of molecules, termed non-coding RNA (ncRNA), plays crucial regulatory roles in shaping cellular activity (46). Accumulating lines of evidence suggest that ncRNAs could initiate pyroptosis in cancers (47). In particular, the regulatory functions and molecular mechanisms of ncRNAs on tumor cell pyroptosis are attracting growing attention with the development of high-throughput sequencing technology (48, 49). In this section, we reviewed the influence of ncRNAs, including microRNA, lncRNA, and circRNA, on tumor pyroptotic cell death (**Figure 1**).

### MicroRNAs

Numerous studies have found that the dysregulation of microRNAs is part of the pathological processes of cancers, including pyroptosis. MicroRNAs bind to a protein-coding mRNA and thereafter decrease target gene expression post-transcriptionally (50). In glioma, miR-214 suppresses cell proliferation and migration *via* caspase-1-mediated pyroptosis. Furthermore, miR-214 bind to the 3’-untranslated region (3’-UTR) of caspase-1 (51). Nevertheless, in cervical cancer, miR-214 targets NLRP3 and thereafter induces pyroptosis in cervical cancer (52). Therefore, in distinct cancers, miR-214 has different targeted genes. Additionally, miR-145/GSDMD signaling is regulated by tanshinone IIA, a lipophilic pharmacologically active compound extracted from *Salvia miltiorrhiza* Bunge (Danshen) (53). Similarly, tanshinone IIA enhances the pyroptosis in nasopharyngeal carcinoma (NPC) and suppresses the progression of NPC *via* miR−125b/foxp3/caspase−1/GSDMD signaling (54). Moreover, in enterovirus A71 (EV-A71)-infected human neuroblastoma, miR-195 inhibits pyroptosis by targeting NLRX1. Therefore, these mechanisms by which microRNAs initiate pyroptosis in cancers provide the antitumor therapy with a novel direction.

### LncRNAs

In addition to mRNAs, complementarity between lncRNA and microRNA has been identified to be part of the regulation of pyroptosis. In colorectal cancer (CRC), lncRNA nuclear paraspeckle assembly transcript 1 (NEAT1) enhances the expression of GSDME and then promotes pyroptosis in CRC by binding to miR-448 (55). Similarly, lncRNA small nucleolar host gene 7 (SNHG7) expression was increased in hepatocellular carcinoma (HCC). Furthermore, SNHG7 inhibited NLR family pyrin domain containing 3 (NLRP3)-dependent pyroptosis serving as a competing endogenous RNA of miR−34a (56). In oral squamous cell carcinoma (OSCC), LINC00958 decreases the levels of miR-4306 to activate the AIM2-dependent pyroptosis (29). In ovarian cancer (OC), lncRNA HOTTIP significantly enhanced NLRP1 inflammasome-mediated pyroptosis *via* the miR-148a-3p/AKT2 axis (57). Therefore, lncRNAs targeting microRNAs are the critical regulators of pyroptosis in cancers and have the potential of therapeutic targets.

Apart from sponging microRNA, lncRNA could influence gene expression directly. For example, in NSCLC, lncRNA X inactive-specific transcript (LNCXIST) suppressed pyroptosis by inhibiting the SOD2/ROS signal pathway (27). In ovarian cancer, long noncoding RNA growth arrest-specific transcript 5 (lncRNA GAS5) functions as a suppressor of tumor progression by inducing inflammasome formation (58). Meanwhile, lncRNA GAS5 blocks the upregulation of gene expression by activating glucocorticoid receptor (GR) (59, 60). Furthermore, the activated GR complex could increase the level of anti-inflammatory proteins in the nucleus or inhibit the proinflammatory proteins in the cytosol (58).

Collectively, lncRNAs initiate pyroptosis in TME by acting as competing endogenous RNAs (ceRNAs) indirectly or influencing targeting gene expression directly.

### CircRNAs

Circular RNAs are a group of different non-coding RNAs with a covalently closed loop structure (61). CircRNAs are abundantly expressed in tumor cells and initiate the pyroptosis-related gene expression (62, 63). On the one hand, circRNAs initiated DNA methylation of pyroptosis-associated genes (64). For example, in glioma cells, circRNA-0001836 promotes the expression of NLRP1 *via* DNA demethylation, leading to the activation of pyroptosis (30). *In vivo*, knockdown of circRNA-0001836 suppressed tumorigenesis by activating NLRP1/GSDMD signaling (30). On the other hand, circRNAs unlock the inhibitory effects of microRNAs on downstream targets serving as microRNA sponges (65–67). For instance, in lung adenocarcinoma (LUAD), circNEIL3 initiates pyroptosis by directly binding to miR-1184 (68). However, the mechanisms by which circRNAs are sensed in the pyroptotic pathway are still vague, which needs to be further explored in the future.

In summary, microRNA, lncRNA, and circRNA initiate cancer cell pyroptosis by sponging upstream or downstream molecules. Understanding these mechanisms has pronounced effects on the development of diagnostic biomarkers and therapeutic targets in clinics. In the future, screening the comprehensive spatial and temporal RNA-expressing profiles of each cancer is needed to expand the utilization of pyroptosis for cancer therapy.

## The influences of pyroptosis-related ncRNAs on tumor resistance

Although overall survival has been prolonged significantly with various treatments, tumor resistance remains an obstacle in clinics (69). Tumor resistance is complex and multifaceted. In 2020, David M. Hyman defined and separated the key determinants of tumor resistance, including tumor burden, growth kinetics, tumor heterogeneity, physical barriers, undruggable cancer drivers, application of therapeutic pressures, immune system, and microenvironment (70). Pyroptosis, an inflammatory PCD, has attracted notable interest within the cancer research community for its potential to address tumor resistance by remodeling immunosuppressive TME.

Recently, it has been illustrated that ncRNAs may regulate tumor resistance *via* pyroptosis (71, 72). For example, lncRNA X inactive-specific transcript (LNCXIST) is upregulated in cisplatin [cis-diamminedichloroplatinum (II); DDP]-treated NSCLC. *In vitro*, LNCXIST knockdown enhances DDP chemosensitivity *via* pyroptosis (71). Furthermore, LNCXIST binds to the TGF-β effector SMAD2, inhibiting its translocation to nucleus. The SMAD2 in nucleus prevented the transcription of NLRP3 (71). Eventually, the DDP chemoresistance is promoted by LNCXIST *via* pyroptosis. *In vivo*, similarly, LNCXIST mediates DDP chemoresistance by inhibiting pyroptosis (71). Additionally, the expression of miR-556-5p is significantly upregulated in the cisplatin-resistant NSCLC (CR-NSCLC) than the cisplatin-sensitive NSCLC (CS-NSCLC) (28). Knockdown of miR-556-5p triggered pyroptotic cell death in cisplatin-treated CR-NSCLC cells *via* upregulating NLRP3 (28). Moreover, the expression of lncRNA ADAMTS9-AS2 was downregulated, and miR223-3p was upregulated in cisplatin-resistant gastric cancer (CR-GC) (73). Furthermore, the overexpression of lncRNA ADAMTS9-AS2 enhanced the cytotoxic effects of cisplatin on CR-GC with the upregulation of NLRP3 inflammasome through targeting miR-223-3p (73). Therefore, these identifications suggest that the expression of LNCXIST, lncRNA ADAMTS9-AS2, miR-556-5p, and miR-223-3p may serve as promising biomarkers to predict DDP treatment efficacy, and may help in the design of new therapies to circumvent DDP chemoresistance in NSCLC and other tumor types.

## NcRNAs remodel tumor microenvironment *via* pyroptosis

The ncRNA-regulated pyroptosis releases various cellular contents, such as inflammatory caspase, IL-1 family, cleaved GSDMD, and chemokines (74), most of which must not be liberated normally. These cellular contents cause unexpected reactions in TME.

### The effects of pyroptosis-related ncRNAs on chronic inflammation

The chronic inflammation caused by ncRNA-regulated pyroptosis promotes tumorigenesis (75), progression, angiogenesis, and metastasis (76–78). For example, in oral squamous cellular carcinoma (OSCC), LINC00958 activates AIM-mediated pyroptosis pathway, which released caspase-1 and IL-1 family into TME and eventually caused chronic inflammation in TME (29). Moreover, the tumor-promoting effects of inflammation were confirmed in the CANTOS trial, in which anti-IL-1 antibody significantly prolonged overall survival and reduced the incidence of lung cancer (79). Therefore, the ncRNA-regulated pyroptosis may have strong tumor-promoting effects in the early stage of tumors.

### The effects of ncRNAs on antitumor immunity

**Figure 2:**
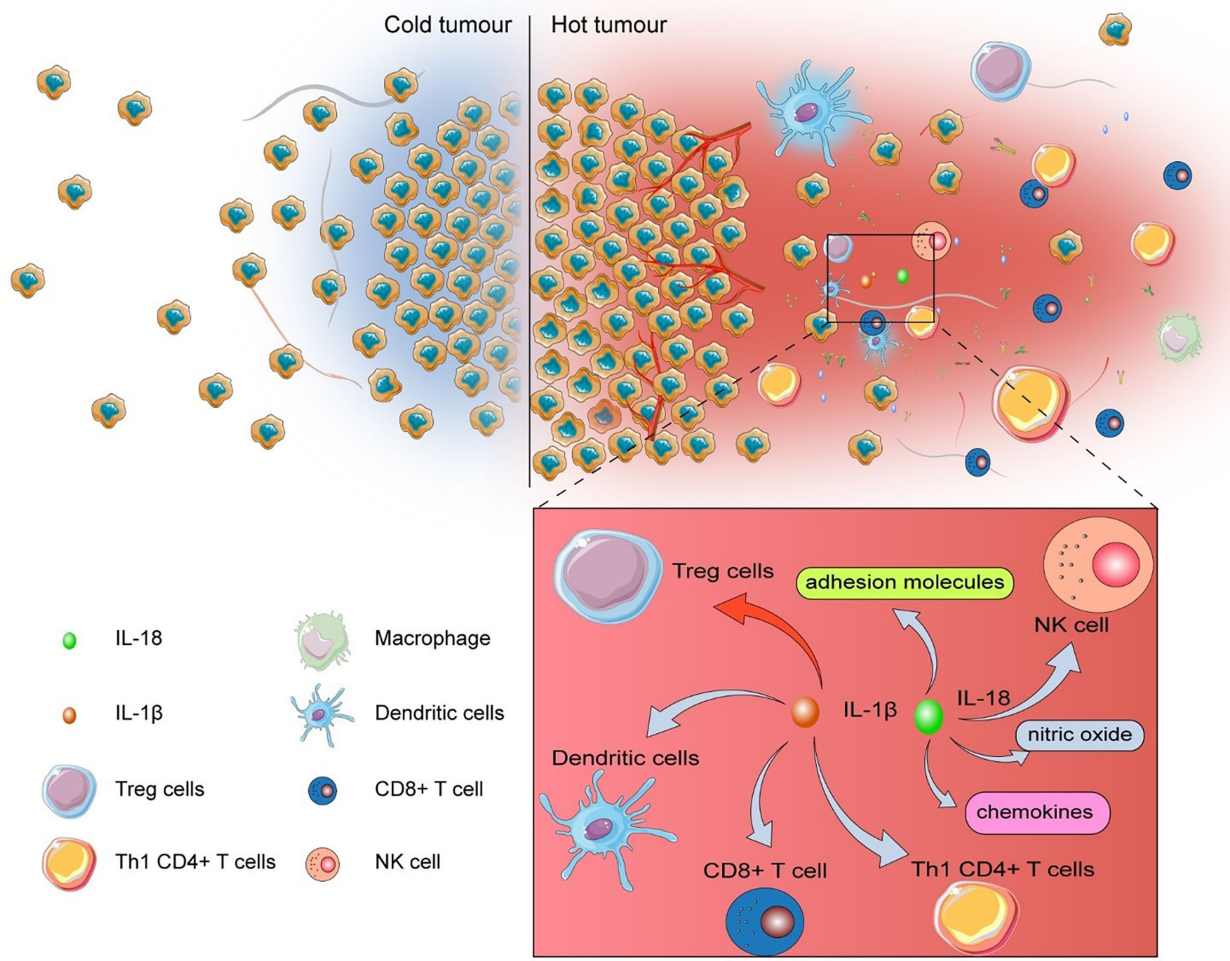
The influence of pyroptosis on TME.After being released into TME, IL-1β escalates the maturation of dendritic cells (DCs), activates antigen-specific cytotoxic CD8+ T cells, recruits Th1 CD4+ T cells, but suppresses the differentiation of immunosuppressive Treg cells. IL-18 polarizes Th1 cells, recruits and activates natural killer (NK) cells, and produces adhesion molecules, chemokine, and nitric oxide.

The tumor with low levels of tumor-infiltrating lymphocytes (TILs) was named “cold tumor”, which is considered as non-responsive to immune checkpoint inhibitors (ICIs) (80). In addition to “cold tumor”, many tumors possess acquired resistance to ICI therapy (80), which remarkably constrains the efficiency of ICIs. MiR-214 elicits robust antitumor immune response in TME *via* promoting pyroptosis with the elevation of IL-1β, IL-18, and caspase-1 (51). After being liberated into TME, IL-1β promotes the maturation of dendritic cells (DCs), activates antigen-specific cytotoxic CD8+ T cells, recruits Th1 CD4+ T cells, and represses the differentiation of immunosuppressive Treg cells (81, 82). Additionally, IL-18 polarizes Th1 cells, recruits and activates natural killer (NK) cells, and produces adhesion molecules, chemokine, and nitric oxide (83) (**Figure 2**).

Therefore, these inflammatory cytokines and caspase not only cause the innate immune response, but also recruit adaptive immune cells, promote antigen presentation, and activate toll-like receptor (TLR) (82, 84, 85). Consequently, ncRNA-regulated pyroptosis enhances the efficiency of immunotherapy. In the future, understanding the underlying mechanisms of pyroptosis-produced inflammatory mediators in TME and the methods to manipulate the inflammatory condition *via* ncRNA-regulated pyroptosis is critical to increase the clinical effect of ICIs on patients.

Collectively, ncRNA-regulated pyroptosis not only converts “cold tumor” to “hot tumor”, but also unleashes various inflammatory mediators to reboot immune response in TME. Therefore, screening ncRNA-expression profiles spatiotemporally is meaningful to expand the clinical application of pyroptosis.

## The clinical value of ncRNAs in pyroptotic cancer cells

Numerous studies have verified that ncRNAs are involved in cell pyroptosis and regulate the content of TME. Hence, converting these studies to clinics is rational and feasible.

### Pyroptosis-associated ncRNA as diagnostic and immunotherapeutic biomarkers

Liquid biopsy is characterized by minimal invasion, which is non-invasive and accessible for cancer diagnosis and monitoring (86). As the mechanisms by which ncRNAs initiate the pathogenesis of cancers were found, the potential diagnostic biomarkers have been emerging. In ovarian cancer, the levels of lncRNA GAS5 was downregulated, while lncRNA HOTTIP was upregulated, indicating that lncRNA GAS5 and HOTTIP may be the futuristic diagnostic biomarkers (57, 58). Similarly, the expression of lncRNA RP1−85F18.6 and ΔNp63 increased and GSDMD cleavage was suppressed in primary CRC, which suggests that the levels of lncRNA RP1−85F18.6, ΔNp63, and GSDMD-N domain should be highlighted diagnostically. Additionally, cancer immunotherapy has gained success in prolonging the survival of patients. However, only a small proportion of patients achieved satisfactory clinical outcomes after immunotherapy (87). Therefore, identifying more precise biomarkers in liquid biopsy for predicting the efficiency of immunotherapy is urgent. For example, it was observed that LNCXIST (27, 71) and miR-556-5p (28) were upregulated in NSCLC, whereas pyroptosis was suppressed. The pyroptosis-generated inflammation evokes a switch from cold tumor to hot tumor, which promotes immunotherapy response. Therefore, LNCXIST and miR-556-5p are the feasible biomarkers for predicting whether patients with NSCLC could respond to immunotherapy effectively.

### Pyroptosis-associated ncRNAs as novel chemo- and immunotherapeutic direction

Tumor resistance has significantly constrained the efficiency of current drugs such as cisplatin (28, 73), sorafenib (88, 89), and pembrolizumab (90, 91). One way to overcome tumor resistance is to develop novel anticancer drugs. For instance, in glioma, the validity of circ_0001836 (30) and caspase-1 (51) serving as therapeutic targets for the treatment of gliomas has been proven. Because there is a conserved binding site for miR-214 in the 3’UTR of the caspase 1 gene. By binding to the the 3’UTR, miR-214 could inhibit cell proliferation and migration in glioma. Meanwhile, circ_0001836 ablation suppresses tumorigenesis in the xenograft model by triggering pyroptosis *via* epigenetically upregulating NLRP1. Nevertheless, the innovation, design, and validation of a new anticancer drug require extraordinary investment and effort. Therefore, natural products have been reviewed, and it has been identified that natural products are a type of potent noncanonical cell death inducers in antitumor therapy (92). For example, tanshinone IIA, a major component of *S. miltiorrhiza*, suppresses cell proliferation and promotes pyroptosis in nasopharyngeal carcinoma by targeting the miR-125b/foxp3/caspase-1 axis, and it is believed that tanshinone IIA is a potent molecule to exert anticancer effects (53, 54). Additionally, in cervical cancer, tanshinone II A was found to regulate tumor growth *via* the miR-145/GSDMD signaling pathway. The expression of GSDMD and miR-145 was dramatically increased after tanshinone II A administration (53). However, one big obstacle to utilizing ncRNAs clinically is the precise delivery of ncRNAs to specific tissues (93). Accumulating lines of evidence have demonstrated that engineered exosome is expected to be a new generation of bioinspired nanoscale drug delivery systems (DDS), which could release chemotherapeutic drugs, mRNAs, regulatory ncRNAs, lipids, and proteins specifically (94–96). For example, engineered exosomes loaded with miR-449a specifically suppress the growth of homologous NSCLC *in vitro* and *in vivo (* 97). Although there are no reports about the engineered exosomes’ loaded ncRNAs regulating pyroptosis in cancers, engineered exosomes could act as DDS to modulate pyroptosis in tumor sites precisely. With the precise delivery platforms, pyroptosis-associated ncRNA drugs have the ability to attain stronger anticancer effects and less unexpected side effects. Therefore, pyroptosis-related ncRNAs offer clinicians a novel direction for either developing new drugs or identifying potent natural products.

Collectively, the tissue-specific characteristics of ncRNAs make it possible to diagnose primary cancer and predict the efficiency of immunotherapy. Engineered exosomes loaded with pyroptosis-related ncRNAs may manipulate tumor-specific pyroptosis for chemo- and immunotherapy precisely.

## Conclusion

Taken together, this article reviews the effects of pyroptosis-associated ncRNAs in cancers. Furthermore, we summarize the mechanisms by which ncRNAs initiate pyroptosis. Meanwhile, we outline the influence of ncRNAs on tumor resistance and TME *via* pyroptosis. Finally, the promising biomarkers and therapeutic targets of pyroptosis-associated ncRNAs are discussed. In the future, the increasingly new advanced analytical techniques and nanotechnology are expected to provide new insights into ncRNA-based biomarkers and drugs.

However, the influence of ncRNA-regulated pyroptosis on tumor resistance and microenvironment is complex. There still exist many problems about the regulation of ncRNAs on pyroptosis, for example, whether there are other ncRNAs that initiate pyroptosis and how ncRNA-regulated pyroptosis modulates tumor resistance and immunity spatiotemporally. Additionally, it is also vague what leads to the expression change of pyroptosis-associated ncRNAs within a cancerous context and how circRNAs are sensed in the pyroptotic pathway. In the future, more experiments are needed to resolve the above questions.

## Author contributions

MZ finished the manuscript and designed the figures. PD collected the related papers. YL, BQ, and ZS gave constructive guidance and made critical revisions. All authors contributed to the article and approved the submitted version.

## Funding

This study was supported by the National Natural Science Foundation of China (81972663, 82173055, and U2004112), the Excellent Youth Science Project of Henan Natural Science Foundation (212300410074), the Key Scientific Research Project of Henan Higher Education Institutions (20A310024), the Youth Talent Innovation Team Support Program of Zhengzhou University (32320290), the Provincial and Ministry co-constructed key projects of Henan Medical Science and Technology (SBGJ202102134), the Key Scientific and Technological Research Projects of Henan Provincial Department of Science and Technology (212102310117), the Henan Provincial Health Commission and Ministry of Health Co-construction Project, and the Henan Provincial Health and Health Commission Joint Construction Project (LHGJ20200158).

## Conflict of interest

The authors declare that the research was conducted in the absence of any commercial or financial relationships that could be construed as a potential conflict of interest.

## Publisher’s note

All claims expressed in this article are solely those of the authors and do not necessarily represent those of their affiliated organizations, or those of the publisher, the editors and the reviewers. Any product that may be evaluated in this article, or claim that may be made by its manufacturer, is not guaranteed or endorsed by the publisher.

## Abbreviations

PCDs, programmed cell deaths; GSDMs, gasdermins; TME, tumor microenvironment; ncRNAs, non-coding RNAs; sncRNA, short ncRNA; lncRNA, long ncRNA; circRNA, circular ncRNA; microRNA, microRNA; GC, gastric cancer; NSCLC, non-small cell lung cancer; CRC, colorectal cancer; DAMPs, danger-associated molecular patterns; PAMPs, pathogen-associated molecular patterns; CC, cervical cancer; GR, glucocorticoid receptor; SBE, SMAD-binding elements; IR, ionizing radiation; HDAC, histone deacetylase; ASC, apoptosis-associated speck-like protein; TIL, tumor-infiltrating lymphocytes; ICIs, immune checkpoint inhibitors; TLR, toll-like receptor; DCs, dendritic cells; NK cells, natural killer cells; AEs, adverse effects; APF, antigen-presenting function.
